# COMMUNICATION PREFERENCES FOR DEPRESCRIBING BISPHOSPHONATES IN NURSING HOME RESIDENTS WITH DEMENTIA

**DOI:** 10.1093/geroni/igad104.2403

**Published:** 2023-12-21

**Authors:** Lauren Fasth, Casey Kelley, Cathleen Colón-Emeric, Carolyn Thorpe, Meredith Gilliam, Jennifer Lund, Laura Hanson, Joshua Niznik

**Affiliations:** UNC Eshelman School of Pharmacy, Chapel Hill, North Carolina, United States; University of North Carolina at Chapel Hill, School of Medicine, Chapel Hill, North Carolina, United States; Duke University School of Medicine, Durham, North Carolina, United States; University of North Carolina at Chapel Hill, Eshelman School of Pharmacy, Chapel Hill, North Carolina, United States; University of North Carolina at Chapel Hill, School of Medicine, Chapel Hill, North Carolina, United States; University of North Carolina at Chapel Hill, Gillings School of Global Public Health, Chapel Hill, North Carolina, United States; University of North Carolina at Chapel Hill, School of Medicine, Chapel Hill, North Carolina, United States; University of North Carolina at Chapel Hill, School of Medicine, Chapel Hill, North Carolina, United States

## Abstract

Bisphosphonates are potential targets for deprescribing in nursing home residents with dementia as goals of care change from preventive to palliative. Yet, prescribers lack communication guidance to address deprescribing. We sought to characterize and compare communication preferences of prescribers and family/informal caregivers regarding bisphosphonate deprescribing. We conducted 23 semi-structured interviews with prescribers (12) and caregivers (11). Prescribers and caregivers were asked to provide their impressions of 7 conversation starters for discussing deprescribing bisphosphonates. A qualitative framework analysis was used to identify themes throughout the interviews across the following domains: content of communication, relationship between communicators, and preferred communication styles. Among prescribers, there were 10 physicians and 2 nurse practitioners; most (9) were female and White. Among caregivers, 8 were female, 7 were White, and 5 were Latino/a; most (9) had a post-secondary degree. The most preferred deprescribing conversation starter for prescribers focused on increased risk of adverse effects, while caregivers most preferred a discussion weighing risks versus benefits. The least preferred conversation starter for deprescribing across both groups focused on the extra effort and cost of continuing bisphosphonates. Similarities and differences between prescribers and caregivers regarding important topics for deprescribing conversations were identified, including quality of life, risk versus benefit, and shared decision-making. While caregivers and prescribers agreed on some communication preferences, this study highlights nuanced differences in preferred communication about deprescribing bisphosphonates. The results of this study may contribute to clinical guidance for prescribers and education materials for caregivers to facilitate more effective conversations about deprescribing bisphosphonates.

